# Dynamic functional changes upon thalamotomy in essential tremor depend on baseline brain morphometry

**DOI:** 10.1038/s41598-024-52410-y

**Published:** 2024-01-31

**Authors:** Thomas A. W. Bolton, Dimitri Van De Ville, Jean Régis, Tatiana Witjas, Nadine Girard, Marc Levivier, Constantin Tuleasca

**Affiliations:** 1 Department of Clinical Neurosciences, Neurosurgery Service and Gamma Knife Center, Centre Hospitalier Universitaire Vaudois, 1011 Lausanne, Switzerland; 2 Department of Radiology, Lausanne University Hospital and University of Lausanne (CHUV-UNIL), 1011 Lausanne, Switzerland; 3 Neuro-X Institute, Ecole Polytechnique Fédérale de Lausanne, 1202 Geneva, Switzerland; 4 Department of Radiology and Medical Informatics, University of Geneva, 1202 Geneva, Switzerland; 5 Stereotactic and Functional Neurosurgery Service and Gamma Knife Unit, Assistance Publique-Hôpitaux de Marseille, Centre Hospitalier Universitaire de la Timone, 13005 Marseille, France; 6 Neurology Department, Assistance Publique-Hôpitaux de Marseille, Centre Hospitalier Universitaire de la Timone, 13005 Marseille, France; 7 Department of Diagnostic and Interventional Neuroradiology, Centre de Résonance Magnétique Biologique et Médicale, Assistance Publique-Hôpitaux de Marseille, Centre Hospitalier Universitaire de la Timone, 13005 Marseille, France; 8 Faculty of Biology and Medicine (FBM), University of Lausanne (UNIL), 1015 Lausanne, Switzerland; 9 Signal Processing Laboratory (LTS 5), Ecole Polytechnique Fédérale de Lausanne, 1015 Lausanne, Switzerland

**Keywords:** Neuroscience, Diseases of the nervous system

## Abstract

Patients with drug-resistant essential tremor (ET) may undergo Gamma Knife stereotactic radiosurgical thalamotomy (SRS-T), where the ventro-intermediate nucleus of the thalamus (Vim) is lesioned by focused beams of gamma radiations to induce clinical improvement. Here, we studied SRS-T impacts on left Vim dynamic functional connectivity (dFC, *n* = 23 ET patients scanned before and 1 year after intervention), and on surface-based morphometric brain features (*n* = 34 patients, including those from dFC analysis). In matched healthy controls (HCs), three dFC states were extracted from resting-state functional MRI data. In ET patients, state 1 spatial stability increased upon SRS-T (*F*_1,22_ = 19.13, *p* = 0.004). More frequent expression of state 3 over state 1 before SRS-T correlated with greater clinical recovery in a way that depended on the MR signature volume (*t*_6_ = 4.6, *p* = 0.004). Lower pre-intervention spatial variability in state 3 expression also did (*t*_6_ = − 4.24, *p* = 0.005) and interacted with the presence of familial ET so that these patients improved less (*t*_6_ = 4.14, *p* = 0.006). ET morphometric profiles showed significantly lower similarity to HCs in 13 regions upon SRS-T (*z* ≤ − 3.66, *p* ≤ 0.022), and a joint analysis revealed that before thalamotomy, morphometric similarity and states 2/3 mean spatial similarity to HCs were anticorrelated, a relationship that disappeared upon SRS-T (*z* ≥ 4.39, *p* < 0.001). Our results show that left Vim functional dynamics directly relates to upper limb tremor lowering upon intervention, while morphometry instead has a supporting role in reshaping such dynamics.

## Introduction

Essential tremor (ET) is a prominent, extremely disabling movement disorder affecting approximately 3% of individuals older than 80 years^1^. It is primarily characterized by the presence of upper limb action tremor for at least 3 years^2^, can co-exist with other symptoms such as impairments in executive function and memory, mood disorders or dementia^3,4^, and may also be prodromal to other conditions^5,6^. Given its heterogeneity regarding the involved clinical and etiological factors^7–9^, it has remained elusive whether ET is in fact a syndrome, a specific disease or a family of diseases^1,10,11^.

While primary pathological and subsequent compensatory molecular and morphological brain changes are believed to center around the cerebellum and eventually recruit deeper structures^12^, tremor generation and maintenance crucially involve cerebellar functional interplays within the cerebello-dentato-rubro-olivary-cerebellar^13,14^ and cortico-ponto-cerebello-dentato-thalamo-cortical^2,15^ networks.

The ventro-intermediate nucleus of the thalamus (Vim) is centrally placed within the latter network and is a well-established surgical target for standard deep brain stimulation (DBS) or stereotactic ablation for tremor^16^, which are indicated for the ET patients who cannot tolerate or do not properly respond to commonly prescribed medications^17^. Standard DBS applies high-frequency electrical pulses through deeply implanted electrodes and is considered safe and efficient to treat ET. It enables stimulation interruption or fine-tuning if needed, but comes with inherent risks (*e.g.*, infection) given the required intervention and the reliance on implanted hardware^18,19^. More recently, magnetic resonance-guided focused ultrasound thalamotomy (MRgFUS) generates a lesion by controlled thermocoagulation, and does not necessitate an invasive operation, while providing a risk/benefit balance akin to more established standard surgical approaches^17,20^. Gamma Knife stereotactic radiosurgical thalamotomy is a minimally invasive alternative that uses stereotactic coordinates to target the Vim, avoiding the need for open surgery, while focusing multiple beams of gamma radiations. It is considered a valuable alternative, particularly for patients with medical comorbidities or uneasy with the prospect of an operation, but it has two main limitations: the target site cannot be confirmed intraoperatively, and clinical benefits only appear after a median of three months, and sometimes up to one year after the intervention^21^. Past works have validated radiosurgical thalamotomy as a safe and effective noninvasive surgical strategy in ET^22–25^.

How the brain of ET patients changes following radiosurgical thalamotomy, and which features may serve as pre-interventional predictors of clinical improvement, are important questions to address. Because ET is believed to initially involve cerebellar neurodegeneration and subsequent reorganization mechanisms, leading to later impacts on other brain regions^12^, morphometric analyses have been a natural direction to follow. In one study, the degree of uniformity of cortical thickness (CT) in right inferior parietal and fusiform areas enabled to discriminate between healthy controls (HCs) and ET patients^26^, while in another, broadly lower CT correlated with increased tremor severity^27^. In a previous voxel-based morphometry (VBM) study, we also showed a direct association between right Brodmann area 18 VBM before radiosurgical thalamotomy and upper limb tremor arrest afterwards^28^. Furthermore, in recent work quantifying CT, surface area (SA) and mean curvature (MC) as morphometric descriptors of cortical brain regions (*i.e.*, surface-based morphometry [SBM] analysis), the statistical dependences across them (inferred through Pearson’s correlation coefficient) were compared before and after the thalamotomy. At the whole-brain level, CT and MC became anticorrelated following thalamotomy, an effect mostly driven by the left fusiform and paracentral gyri, left posterior cingulate cortex, right banks superior temporal sulcus and right inferior temporal cortex. On the other hand, SA and MC became correlated, mostly so in the bilateral fusiform gyrus and right inferior temporal cortex^29^. Graph theoretical investigations provided further insight on regional changes, as CT in the right lingual and bilateral rostral middle frontal gyri exhibited lower dependence to MC of the rest of the brain after thalamotomy, while conversely, an increase was seen for the left precentral gyrus^30^.

In reports instead characterizing the functional interplays of the targeted left Vim by computing its resting-state functional connectivity (RS FC) to the rest of the brain, FC to the right insular and orbitofrontal cortices, to the right posterior parietal, supramarginal and inferior frontal gyri, and to the bilateral frontal eye fields decreased to non-significant values upon thalamotomy, while conversely, FC to the right supplementary motor area increased to around zero from an initial anticorrelation^31^. In addition, more negative left Vim FC to the ipsilateral fusiform gyrus pre-intervention correlated with larger clinical improvement^32^.

Despite their valuable insight, the above previous studies suffer from some shortcomings. Given their reliance on Pearson’s correlation coefficient, the conducted SBM investigations could not individually address the effects of cross-property covariance (which would contribute to the numerator of the coefficient) and variance of individual properties (which would instead impact its denominator). In addition, only pairs of morphometric properties were considered in each assessment, falling short of a fully multivariate treatment. As for functional analyses, they operated under the assumption of static cross-regional interplays, even though FC exhibits prominent temporal dynamics with cognitive and clinical relevance^33,34^. In ET, only one study to date has explicitly examined functional brain dynamics and its changes upon thalamotomy^35^, but it focused on co-(de)activations with the right extrastriate cortex. The dynamic interplays of the targeted Vim, and their evolution upon thalamotomy, thus remain unknown.

In the present work, we seek to overcome these limitations in a joint analysis of SBM and RS functional MRI (fMRI) data acquired on a partly overlapping set of patients with ET, scanned before and 1 year after radiosurgical thalamotomy. We address the following questions: (1) *whether thalamotomy induces a renormalization of morphometric and dynamic functional brain properties in ET*, (2) *which pre-thalamotomy features better correlate with clinical recovery*, and (3) *how potential structure/function couplings are impacted by the intervention*.

To answer these questions, we quantify similarity of the pre- and post-thalamotomy ET data points (respectively termed ET_pre_ and ET_post_ from there onwards) to a set of matched HCs. For SBM analysis, we leverage a recently introduced analytical pipeline^36^ to model the HC morphometric data by a multivariate Gaussian, and subsequently quantify the likelihood of ET data points to be issued from this distribution. By this mean, we derive a measure of similarity to HCs. For dynamic FC (dFC) analysis, we employ a sliding-window approach to extract recurring dFC states across time and subjects^37^ from our HC population. Then, ET_pre_ and ET_post_ dFC estimates are matched to these states to quantify spatial similarity and temporal occurrences.

For SBM analysis, given our previous work^29,30^, we expected movement-related and visual regions to renormalize upon radiosurgical thalamotomy. As a Partial Least Squares analysis evidenced a correlation between morphometric similarity to HCs and upper limb tremor involving the paracentral and supramarginal gyri^36^, we deemed these regions most likely to associate with clinical recovery. For dFC analysis, considering past work^38^, we expected the RS fMRI data from HCs to be optimally clustered into a restricted set of dFC states. Based on a previous state-based study of ET^35^, we thought that temporal occurrences would renormalize upon radiosurgical thalamotomy, and potentially correlate with clinical recovery. Having not been investigated beforehand, we had no clear hypothesis regarding spatial similarity. As for cross-modality analysis, we expected to observe a correlation between morphometric and dFC similarity to HCs, and its increase upon intervention.

## Materials and methods

### Subjects

We studied 34 right-handed patients (17 males) with drug-resistant ET. They were 70.06 ± 9.12 years old when initially assessed, and 23 had a familial history of ET. Onset age for the symptoms was 34.53 ± 20.79 years (min: 8, median: 30.5, max: 74), and symptoms’ duration was 35.53 ± 18.28 years (min: 5, median: 33, max: 61). Neurological assessment was performed by T.W., a neurologist specialized in movement disorders. All patients had a clear diagnosis of ET^39^; inclusion criteria are listed in the Supplementary information. No patient was under medication at the time of study or showed structural abnormalities upon 3 T MRI.

Patients were clinically assessed every three months after thalamotomy of the left Vim, which enabled to precisely quantify time to tremor improvement (127.56 ± 81.38 days); and scanned before and 1 year after the intervention, to account for the progressive and delayed clinical effect. The Tremor Score on Treated Hand (TSTH) from the Fahn-Tolosa-Marín rating scale^40^ was used to quantify tremor severity in the patients and its evolution upon intervention. The severity of head tremor was also quantified (Tremor Research Group Essential Tremor Rating Assessment, range: 0–3).

ET patients were compared to a group of 29 age- and gender-matched HCs (69.93 ± 7.14 years old, 12 males). While the full set of ET patients and HCs was used for SBM analysis (based on T1-weighted structural images), only 23 patients contributed at least one RS fMRI scan for dFC analysis, and the recordings of both time points were retained for 13 of them. There were 18 remaining scans both in the ET_pre_ and ET_post_ groups. The RS fMRI data from 14 HCs was also retained. The reason is the exclusion of poor-quality recordings, due to an excessively trimmed field of view or the presence of obvious artefacts when inspecting functional volumes, or to too elevated head movement, as further detailed below (see Data processing subsection). Clinical and demographic data are summarized in Table 1 for the final sets of subjects considered in each type of analysis.

**Table 1:** Demographic and clinical details of the subjects.

Variable	Morphometry analyses					Dynamic functional connectivity analyses				
	HC	ET_pre_	ET_post_	*p*(HC − ET)	*p*(ET_post_ − ET_pre_)	HC	ET_pre_	ET_post_	*p*(HC − ET)	*p*(ET_post_ − ET_pre_)
N	29	34	34			14	18	18		
Age at baseline evaluation [years]	69.93 ± 7.14 [59, 69, 83]	70.06 ± 9.12 [49, 72, 83]		*t*_61_ = − 0.06 *p* = 0.95	1-year increase	70.21 ± 6.8 [61, 69, 81]	69.3 ± 10.1 [49, 71.5, 82]	71 ± 9.68 [51, 72.5, 83]	*t*_30_ = 0.45 *p* = 0.66	*t*_34_ = 0.51 *p* = 0.62
Gender [M:F]	12:17	17:17		χ^2^ = 0.47 *p* = 0.49		5:9	7:11	8:10	χ^2^ = 0.03 *p* = 0.85	χ^2^ = 0.11 *p* = 0.74
TSTH score [points]		20.41 ± 5.53 [8, 20.5, 30]	6.26 ± 7.71 [0, 3, 27]		***t***_**66**_** = − 8.69** ***p* = 1.52 × 10**^**–12**^		19.94 ± 6.21 [8,20.5,30]	5.83 ± 8.79 [0, 2, 27]		***t***_**34**_** = **− **5.57** ***p* = 3.17 × 10**^**–6**^
Head tremor score [points]		1 ± 0.85 [0, 1, 2]	0.59 ± 0.75 [0, 0, 2]*		***t***_**65**_** = **− **2.16** ***p* = 0.035**		0.89 ± 0.83 [0,1,2]	0.41 ± 0.62 [0, 0, 2]*		*t*_33_ = − 1.92 *p* = 0.064
Symptoms’ duration [years]		35.53 ± 18.28 [5, 33, 61]			Same subjects		30.94 ± 16.52 [5,27.5,61]	29.89 ± 16.38 [5, 26, 55]		*t*_34_ = − 0.19 *p* = 0.85
Age of symptoms’ onset [years]		34.53 ± 20.79 [8, 30.5, 74]			Same subjects		38.39 ± 19.13 [11, 35, 74]	41.11 ± 20.36 [13, 38.5, 74]		*t*_34_ = 0.41 *p* = 0.68
Time to tremor improvement [days]		127.56 ± 81.38 [15, 120, 300]**			Same subjects		149.24 ± 87.06 [30,180,300]*	132.47 ± 76.56 [15, 120, 240]*		*t*_32_ = − 0.6 *p* = 0.56
Familial ET? [Y:N]		23:11			Same subjects		8:10	8:10		χ^2^ = 0 *p* = 1
MR signature [ml]		0.12 ± 0.13 [0.002, 0.076, 0.6]			Same subjects		0.12 ± 0.16 [0.002, 0.069, 0.6]	0.15 ± 0.16 [0.014, 0.093, 0.6]		*t*_34_ = 0.53 *p* = 0.6

For healthy controls (HCs) and essential tremor (ET) patients before (ET_pre_) and after (ET_post_) intervention, values are reported as mean ± standard deviation, with minimum, median and maximum into squared brackets. Significant statistical comparisons are highlighted in bold. */**: value not recorded for one/two subject(s)

*M* male, *F* female, *TSTH* tremor score on treated hand, *Y* yes, *N* no.

The Timone University Hospital Ethical Committee (ID-RCB: 2017-A01249–44) granted formal approval for this study (including by the Ethics Committee at national level, CNIL-MR-03). All methods were performed in accordance with the relevant guidelines and regulations. Individual informed consent was obtained from all subjects.

All details regarding data acquisition are provided in the Supplementary information.

### Intervention and one-year MR signature volume assessment

Thalamotomy was performed using Leksell Gamma Knife (LGK, Elekta Instruments, AB, Sweden) between September 2014 and April 2016, at the Centre Hospitalier Universitaire de la Timone (Marseille, France), always by the same neurosurgeon (J.R.). In each case, the Leksell coordinate G frame (Elekta Instruments, AB, Sweden) was applied under local anesthesia. After its positioning, stereotactic computed tomography and MRI were both performed on the patient.

Landmarks of interest, such as the anterior and posterior commissures, were individually identified on an MR scan (T2 CISS/FIESTA sequence, Siemens). Targeting was achieved individually with the Guiot diagram^41^, placed 2.5 mm above the anterior–posterior commissure line and 11 mm lateral to the third ventricle wall. A single 4-mm isocenter was used with a maximum prescription dose of 130 Gy.

There is a specific MR signature of radiosurgical thalamotomy, known to differ across subjects both in terms of aspect (with or without contrast enhancement) and corresponding volume^21^. It was contoured on a T1-weighted Gadolinium-injected scan acquired at one-year follow-up, and usually corresponds to the 90 Gy isodose line. The individual patient’s Gadolinium-injected MR image was imported in the Leksell GammaPlan software (Elekta Instruments, AB, Sweden), and co-registered with the stereotactic imaging. A manual drawing was made for each individual case, on each slice.

### Data processing

#### Surface-based morphometry

*Freesurfer*^42^ version 5.3.0 [https://surfer.nmr.mgh.harvard.edu/fswiki/DownloadAndInstall5.3] was used to extract CT, SA and MC from native structural MR images for a set of *P*_cort_ = 68 cortical regions (see Supplementary information for details). In addition, we also extracted regional volume for *P*_noncort_ = 19 non-cortical areas, including the cerebellum and subcortex, using *Freesurfer*’s automatic subcortical segmentation approach^43^. Supplementary Table 1 summarizes all brain regions considered in our morphometric analyses.

To account for the confounding impacts of age, gender and total grey matter volume in our analyses while handling the fact that two scans were available for ET patients but not for HCs, a mixed-effects model strategy was employed (see Supplementary information for details).

#### Dynamic functional connectivity

A similar preprocessing scheme was applied to each of the functional scans, using SPM12 [https://www.fil.ion.ucl.ac.uk/spm/software/spm12/] for initial steps. Functional volumes were realigned and subsequently co-registered to the T1 structural image. Segmentation was performed to derive the deformation field that was then used to warp fMRI volumes into the common MNI space. T.A.W.B. inspected warped volumes for each scan, ensuring that preprocessing steps until normalization ran properly. This involved checking that the cerebrospinal fluid compartment precisely overlapped with that of an MNI template volume; verifying that the functional signal of the subject’s eyes lied within the orbits seen on the template volume; and ensuring that the functional signal within the brain had identical boundaries to the template ones. Scans with excessively trimmed field of view were discarded. At this stage, a total of 11 HC, 9 ET_pre_ and 13 ET_post_ scans were discarded, primarily because the anterior brain or the cerebellum was too largely trimmed.

Subsequent steps were performed using custom scripts and MATLAB version 2020b (MathWorks, Natick, MA). The first 3 volumes were discarded to enable magnetization equilibration. Then, the voxel-wise data was converted into a restricted set of parcels, combining three different atlases: the Schaefer atlas^44^ (400 regions, 17 networks) for cortical areas, the Tian atlas^45^ (scale 3, 50 regions) for subcortical regions, and the decomposition into 26 subregions from the AAL atlas^46^ for the cerebellum. In total, there were thus 476 areas available, of which 13 were excluded because they were not included in at least one scan owing to a trimmed field of view. Thus, the present work considers dFC of the left Vim with 462 other brain regions, summarized in Supplementary Table 2.

To clean the regional time courses from artefactual signal sources, conservative white matter and cerebrospinal fluid masks from the DPARSF toolbox^47^ were used to compute associated regressors. Together with the 6 head motion parameters obtained at the realignment step and a Discrete Cosine Transform basis for drifts (cut-off: 0.01 Hz), they were regressed out from the data. Finally, framewise displacement (FD)^48^ was computed, and the scans for which more than 30% of frames were corrupted (defined as the frames with FD > 0.5 mm, the frames beforehand and the two frames afterwards) were discarded. Following this step, 4 HC, 3 ET_pre_ and 1 ET_post_ scans were discarded.

From this preprocessed data, sliding-window analysis was conducted. We used a rectangular window with size W = 30 TRs (99.9 s) − *i.e.*, the inverse of the minimal frequency remaining in the data (0.01 Hz)^49^, and step size Δ = 2 TRs (6.6 s). FC within each temporal sub-window was quantified with Pearson’s correlation coefficient, discarding the frames corrupted by head movement. To further guarantee the quality of the analyzed data, only windows for which more than 20 samples remained were retained for analysis, and ET scans for which less than 85 windows remained were discarded. Following this step, 4 ET_pre_ and 2 ET_post_ scans were discarded. DFC data thus remained for 14 HCs, 18 ET_pre_ scans, and 18 ET_post_ scans.

### Data analysis

#### Surface-based morphometry

We computed the log-likelihood of ET_pre_ and ET_post_ data to be issued from the HC distribution as a similarity measure (see Supplementary information for details). It was compared across groups with a rank-sum test, run separately for each region. The obtained *p*-values were Bonferroni-corrected for the number of performed tests (87).

As a supplementary analysis, we assessed how the results would change if only the 23 ET patients contributing to dFC analysis were considered.

For the regions that reached significance in the above assessment, individual mean and (co)variance coefficients were subsequently contrasted across groups through a non-parametric permutation-based significance assessment of each ET_post_ − ET_pre_ difference (100′000 folds, two-tailed testing). The obtained *p*-values were Bonferroni-corrected for the number of performed tests (103).

#### Dynamic functional connectivity

To extract dFC states expressed in our HC population, clean dFC estimates (*i.e.*, windows with at least 20 non-corrupted samples) were concatenated across all 14 subjects. This resulted in 1136 samples, each of dimension 462 (the number of connections to the left Vim).

To define an optimal number of states, we used consensus clustering^50^: over 200 folds, 80% of samples were randomly selected, and *K*-means clustering was performed (cosine distance) for *K* ranging from 2 to 20. The percentage of ambiguously clustered pairs (PAC)^51^ was computed as a measure of clustering quality, and clearly revealed *K* = 3 as an optimum (Supplementary Fig. 1). A final *K*-means clustering step was then performed (300 folds, 200 iterations each), yielding *K* = 3 dFC states characteristic of the HC population.

The first 85 dFC estimates from each available ET scan were then matched to these dFC states through Pearson’s correlation coefficient. The most similar state to a given dFC estimate was assumed to be expressed at that time point (*i.e.*, winner-takes-all approach), enabling the computation of temporal occurrences (number of times a given state is expressed) for each scan. In addition, the mean and standard deviation of spatial similarity to the expressed state were computed, and the coefficient of variation was derived as a measure of spatial stability of state expression. Temporal occurrences (*K* values per scan), and each spatial stability measure (*K* values per scan as well), were considered as metrics of interest in our analyses.

#### Quantification of the impact of thalamotomy

To determine whether a given metric differed between the pre- and post-thalamotomy stages, a two-way ANOVA was conducted, using group label (ET_pre_ and ET_post_) as a fixed effect and subject label as a random effect. The model takes the form:

$$M_{g,s} = \mu + \alpha_{g} + \beta_{s} + \varepsilon_{g,s} .$$

In the above equation, *M*_*g*,*s*_ is the value of the metric at hand for subject *s* in group *g*, μ is the global mean across both groups, α_*g*_ is the fixed group effect, *β*_*s*_ is the random subject effect, and ε_*g*,*s*_ is the error term. The random effect is assumed to follow a normal distribution with mean 0 and variance σ_β_^2^, and the error follows a normal distribution with mean 0 and variance σ_ε_^2^. A significant fixed group effect means that the null hypothesis of equal means across groups can be rejected (that is, there is an impact of thalamotomy at the group level). A significant random subject effect means that the null hypothesis σ_β_^2^ = 0 can be rejected (in other words, different subjects show different extents of expression of the metric at hand, meaning that there is a large heterogeneity across subjects).

We evaluated temporal occurrences and spatial stability of the expressed dFC states for potential group differences (4* K* features). Obtained *p*-values were Bonferroni-corrected for the number of states (*K*), and significance level was set at α = 0.01.

#### Extraction of predictive pre-thalamotomy features

To establish whether a given metric before thalamotomy correlates with clinical improvement, we considered the difference in TSTH score (ΔTSTH = TSTH_pre_—TSTH_post_) as outcome measure, so that a more positive value indicates greater recovery, and the following general linear model:

$$\begin{aligned} \Delta {\text{TSTH}} & = \beta_{0} + \beta_{1} A_{s} + \beta_{2} G_{S} + \beta_{3} M_{s} + \beta_{4} R_{s} + \beta_{5} S_{s} + \beta_{6} F_{s} + \beta_{7} H_{s} \\ & \quad + \beta_{8} \left( {M_{s} \times R_{s} } \right) + \beta_{9} \left( {M_{s} \times S_{s} } \right) + \beta_{10} \left( {M_{s} \times F_{s} } \right) + \beta_{11} \left( {M_{s} \times H_{s} } \right) \\ \end{aligned}$$

Importantly, in addition to modelling the impacts of age *A* and gender *G* for subject *s*, we also consider that of the radiological signature *R*, as well as its interaction with the metric *M*. This is because previous work has shown that the MR signature correlates with pre-interventional functional brain features^52^. Furthermore, we include symptoms’ duration (*S*), the presence of a familial history of ET (*F*, nominal variable with 0 = no and 1 = yes), and head tremor intensity (*H*) as regressors, as well as their interactions with *M*, to examine whether the clinical heterogeneity that these factors entail may interact with the probed brain marker in associating with clinical recovery.

In addition to the above, we performed a similar analysis taking time to tremor improvement as the dependent variable, as it could also be that clinical recovery occurs at different paces as a function of brain markers.

Note that for the assessments performed on morphometric features, since age and gender were already regressed out during processing, these two covariates were omitted from the model. Significance level was set at α = 0.01.

#### Similarity across modalities

To compare SBM and dFC data, we considered the log-likelihood to be issued from the HC distribution (for SBM analysis, *P* values per subject), and the mean similarity to the dFC states (for dFC analysis, *K* values per subject). Pearson’s correlation was computed to yield a *P* x *K* matrix of similarity values for the ET_pre_ group, and another for the ET_post_ group.

For each state, the distribution of correlation values with the whole brain was contrasted between before and after thalamotomy through a rank-sum test. The resulting *p*-values were Bonferroni-corrected for *K* = 3 states, and significance level was set at α = 0.01.

### Previous data uses and implementation details

The morphometric data analyzed therein was already examined in two previous structural covariance analysis studies^29,30^, which performed pair-wise assessments of cross-property dependences and did not discriminate between property variance and cross-property covariance. The analytical framework leveraged in the present work was applied on ET_pre_ morphometric profiles to assess their differences to HCs^36^, but never to the ET_post_ data.

A subpart of the functional data considered here was already analyzed in a series of studies addressing the specificities of ET and the impacts of thalamotomy^31,32,35,52–54^. However, none of these previous reports examined dFC of the left Vim, and only one focused on temporal dynamics in ET^35^.

Colormaps for plotting were generated with the *cbrewer* toolbox. Surface visualizations of brain patterns were created using *BrainNet*^55^.

All the scripts used in this work are freely available at https://github.com/TiBiUan/DYNET_Analysis.git.

## Results

### Dynamic functional connectivity states extracted from HCs

The dynamic evolution of FC in HCs could be disentangled into *K* = 3 separate dFC states (Supplementary Fig. 1), as seen from a clear PAC global minimum and upon inspection of the consensus matrices. State 1 occurred in 419 (36.88%) frames and was expressed 36.38 ± 36.95% of the time in individual subjects. State 2 only occurred in 182 (16%) frames (16.47 ± 35.78% of the time per subject). State 3 occurred the most (535 or 47.1% of frames, 47.15 ± 37.8% of the time subject-wise).

**Figure 1:**
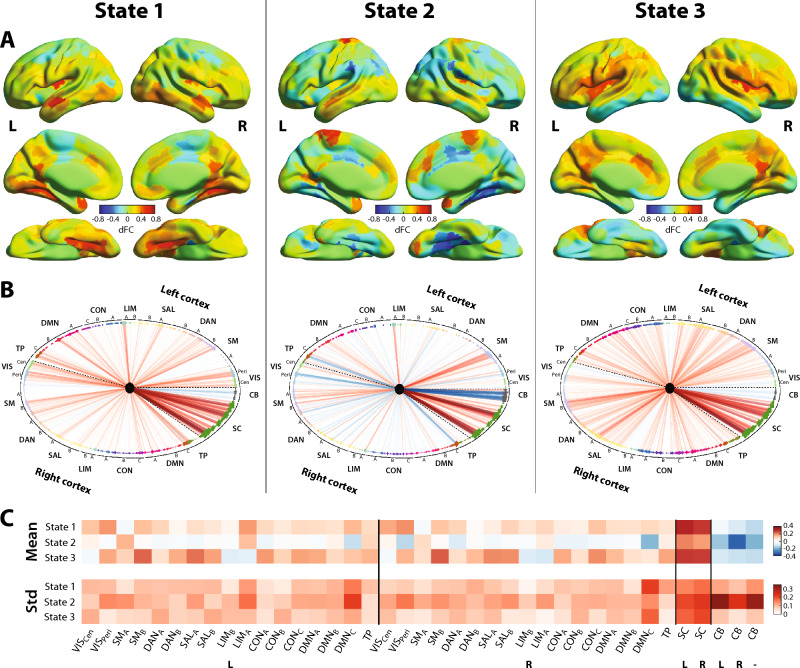
Three dynamic functional connectivity states expressed by healthy controls. Surface representation (**A**) and circular plot (**B**) of all three states in terms of dFC with the left Vim. For surface images, color denotes dFC strength and only cortical areas are represented. For circular plots, red/blue edges denote positive/negative-valued dFC, and edge width is proportional to dFC strength. Brain regions are arranged anticlockwise, starting from the far right of the representation, and dashed lines separate left and right cortical regions, as well as subcortical/cerebellar areas. Node color depicts network assignment^44^, and squares/diamonds symbolize left/right hemispheric regions (stars depict vermis cerebellar areas). (**C**) For all investigated networks (columns) and states (rows), mean and standard deviation across regions. L: left, R: right, VIS: visual network, SM: somatomotor network, DAN: dorsal attention network, SAL: salience network, LIM; limbic network, CON: control network, DMN: default mode network, TP: temporo-parietal network, SC: subcortical network, CB: cerebellar network, Cen: central, Peri: peripheral, Std: standard deviation.

In terms of spatial properties (Fig. 1; see also Supplementary Fig. 2 for a word cloud visualization of the same information), all states showcased their largest positive-valued dFC (*i.e.*, correlated activity with the left Vim) with other subcortical areas, as well as negative-valued dFC (anticorrelated activity with the left Vim) with cerebellar regions. A more restricted set of subcortical areas, and stronger cerebellar anticorrelations, were observed for state 2. Regarding interplays with the cortex, states 1 and 3 primarily featured positive-valued dFC, while the pattern was more mixed for state 2. Only state 1 showed correlations with the full visual network, while state 2 displayed (anti)correlations with specific peripheral visual regions (defined as per Schaefer et al., including extra-striate inferior and superior as well as striate calcarine cortices), and state 3 mostly included positive-valued dFC with these peripheral visual regions. Primary somatomotor areas were only largely correlated with the left Vim in state 2, as opposed to higher-level somatomotor areas for the two others. Dorsal attention, salience and control networks exhibited a gradient of positive-valued dFC across states, strongest in state 3 and weakest in state 2. For the default mode network, state 3 exhibited the most widespread correlations, but the strongest connections were found in state 1. Finally, state 1 also showcased strong temporo-parietal dFC.

### Changes in ET patients’ dFC state metrics upon thalamotomy

**Figure 2:**
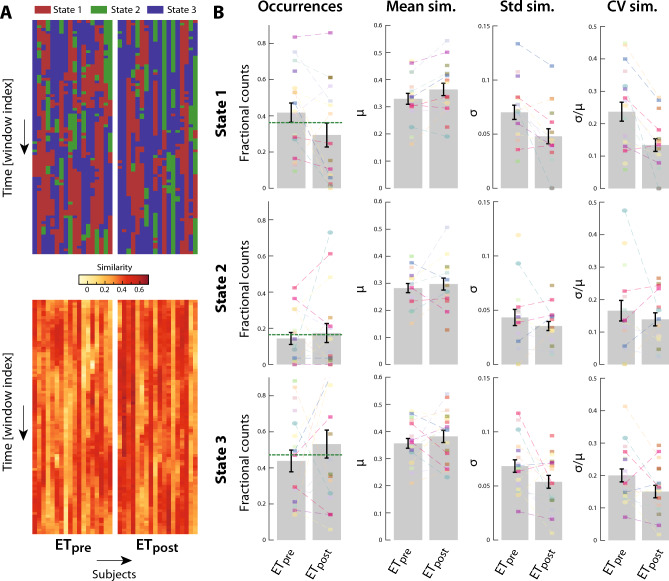
Evolution of dynamic functional connectivity metrics in essential tremor patients upon thalamotomy. (**A**) (Top) State assignment for ET frames (before/after thalamotomy: left/right) across subjects (columns) and over time (rows). (Bottom) In a similar representation, evolution of spatial similarity of the dynamic functional connectivity estimates to their assigned state. (**B**) For all three states (rows) and four investigated metrics (columns), values across subjects before (ET_pre_) and after (ET_post_) thalamotomy. Bars denote the average, error bars depict standard error of the mean, and colored data points stand for individual subject values. Dashed lines connect the data points that belong to the same patient, and the horizontal dashed green lines for occurrences bar plots denote the average in the healthy controls. Mean sim.: mean similarity, Std sim.: standard deviation of similarity, CV sim.: coefficient of variation of similarity.

Matching of the ET_pre_ and ET_post_ windowed dFC estimates to these states (Fig. 2A) revealed that they were all also expressed in the ET patient population, in a way that differed along time and across subjects. Furthermore, the spatial similarity of dFC estimates to their assigned state also fluctuated along the same dimensions. We thus examined temporal occurrences and spatial similarity features to assess whether there was an impact of thalamotomy, including a random subject effect to account for multiple measurements in only a subset of patients (Fig. 2B). The analysis revealed no group difference for temporal occurrences (all *p*-values > 0.1). For mean spatial similarity, there was a significant random subject effect for state 1 (*F*_1,22_ = 7.89, *p* = 0.003), indicating strong differences between individual patients with this measure. The coefficient of variation of spatial similarity, which jointly accounts for mean and standard deviation impacts, yielded a significant fixed effect for state 1 (*F*_1,22_ = 19.13, *p* = 0.004, η^2^ = 0.166), with a decrease observed upon thalamotomy. Thus, significant spatial renormalization was observed in terms of state 1 expression.

### Links between pre-thalamotomy dFC metrics and clinical recovery

Investigation of clinical predictive potential revealed a significant relationship between clinical improvement (quantified as the drop in TSTH) and the standard deviation of similarity to state 3 (*t*_6_ = − 4.24, *p* = 0.005, Cohen’s *d* = − 1.00 ± 0.91, 99% confidence interval), a significant interaction with family history of ET (*t*_6_ = 4.14, *p* = 0.006, Cohen’s *d* = 0.98 ± 0.91), and a significant effect of head tremor (*t*_6_ = − 4.15, *p* = 0.006, Cohen’s *d* = − 0.98 ± 0.91). Hence, clinical recovery is greater when state 3 is expressed with less variability, and this relationship is more strongly present in patients without a familial history of ET.

In addition, there was a significant MR signature-by-metric interaction for state 3 temporal occurrences (*t*_6_ = 3.76, *p* = 0.009, Cohen’s *d* = 0.89 ± 0.9). As the same term was close to significance for state 1 with opposite sign (*t*_6_ = − 2.32, *p* = 0.059, Cohen’s *d* = − 0.55 ± 0.87), we probed the difference between state 1 and 3 counts (Counts_3_ − Counts_1_) in a follow-up analysis: the interaction remained significant (*t*_6_ = 4.6, *p* = 0.004, Cohen’s *d* = 1.08 ± 0.92), indicating that the more state 3 is expressed over state 1, the better the recovery, in a way that also depends on the MR signature. Similar results were obtained when a more restricted set of regressors (metric, MR signature volume, age, and gender) was used (see Supplementary information).

When time to tremor improvement was considered as dependent variable instead, no significant association was found.

### Morphometric changes upon thalamotomy

**Figure 3:**
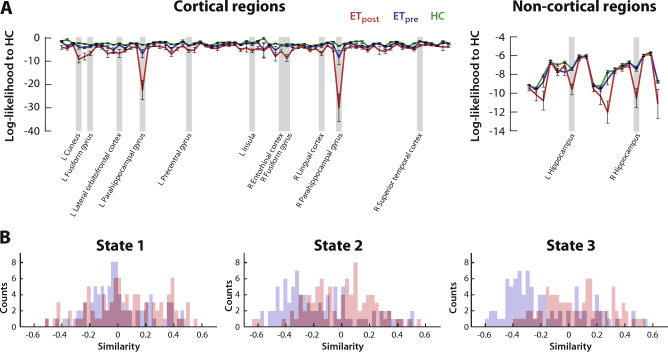
Morphometric heterogenization upon intervention, and state-specific disappearance of cross-modality anticorrelations. (**A**) For cortical (left) and non-cortical (right) regions, log-likelihood to be issued from the HC distribution for the data points of the HC group (green), ET_pre_ group (blue) and ET_post_ group (red). Regions for which the ET_pre_ *versus* ET_post_ log-likelihood difference reached significance are highlighted in light grey. (**B**) For each state, histogram of similarity values (log-likelihood to be issued from the morphometric HC distribution correlated to average spatial similarity of dFC state expression) across regions before (blue) or after (red) thalamotomy.

On morphometric data, we quantified the log-likelihood for a regional ET estimate to be issued from the HC distribution as a measure of similarity (Fig. 3A). The average log-likelihood across subjects was consistently larger before than after thalamotomy, a difference significant in 11 cortical regions: the bilateral fusiform (ET_post_-ET_pre_ group difference: *z* = -4.02, *p* = 0.005, η^2^ = 0.47 and *z* = − 4.16, *p* = 0.003, η^2^ = 0.51, respectively left and right sides) and parahippocampal (*z* = − 4.84, *p* = 0.0001, η^2^ = 0.69 and *z* = − 5.75, *p* < 10^–5^, η^2^ = 0.97) gyri, left cuneus (*z* = − 4.02, *p* = 0.005, η^2^ = 0.47), lateral orbitofrontal cortex (*z* = − 4.12, *p* = 0.003, η^2^ = 0.5), precentral gyrus (*z* = − 3.81, *p* = 0.012, η^2^ = 0.43) and insula (*z* = − 3.66, *p* = 0.022, η^2^ = 0.39), right entorhinal cortex (*z* = − 3.78, *p* = 0.013, η^2^ = 0.42), lingual cortex (*z* = − 3.66, *p* = 0.022, η^2^ = 0.39) and superior temporal cortex (*z* = − 3.67, *p* = 0.021, η^2^ = 0.4). There were also two significant subcortical areas: the bilateral hippocampus (*z* = − 4.48, *p* = 0.0006, η^2^ = 0.59 and *z* = − 4.03, *p* = 0.005, η^2^ = 0.48). In sum, there was thus no evidence for morphometric renormalization upon thalamotomy.

More details regarding investigations at the level of individual mean and (co)variance coefficients can be found in the Supplementary information. Additionally, when only the subjects contributing to dFC analysis were considered, a similar but more restricted set of regions were pinpointed, owing to the lower statistical power of the analyses (see Supplementary information for details).

### Links between pre-thalamotomy SBM metrics and clinical recovery

There was no link between pre-intervention log-likelihood in any of the regions that showed significance and clinical recovery (all *p*-values > 0.1).

### Cross-modality coupling

Finally, we quantified the correlation between the mean spatial similarity in dFC state expression and the morphometric similarity to HCs (Fig. 3B; see also Supplementary Fig. 4) in a cross-modality analysis. For state 1, the distributions of correlation values were similarly centered around 0 both before and after thalamotomy (rank-sum test for ET_post_ – ET_pre_ group difference: *z* = 1.89, *p* = 0.058, η^2^ = 0.041). However, for states 2 and 3, we observed a broad pattern of anticorrelation before thalamotomy, which disappeared afterwards (respectively *z* = 4.39, *p* = 1.16 × 10^–5^, η^2^ = 0.22 and *z* = 5.83, *p* = 5.54 × 10^–9^, η^2^ = 0.39). In other words, before thalamotomy, a patient who showed greater average spatial conformity to HC dFC states 2 and 3 also tended to show less similarity to the regional HC morphometric profiles, but this relationship disappeared after thalamotomy.

## Discussion

### The left Vim: a dynamic cornerstone of the tremor network

In this study, we observed that the left Vim reconfigures its functional interactions with the rest of the brain in HCs over the course of an RS fMRI scanning session, showcasing the recurring expression of three different dFC states (Fig. 1, Supplementary Fig. 2). We also showed that patients with ET express these states with subject-specific temporal occurrences and stability in spatial similarity, in a way that is partly modulated by thalamotomy (Fig. 2).

Correlated activity with other subcortical areas, anticorrelations with cerebellar regions, and broad connectivity patterns with cortical networks, were hallmarks of all three states. This is consistent with known anatomical connections, as the Vim is linked to the cerebellum through the dentate nucleus, and to the motor cortex^1,2^. Furthermore, there are known projections from the thalamus (including its ventral posterior subpart, studied here) to the basal ganglia^56^. A possible explanation for the existence of anticorrelations could be that cerebellar activation induces the locking of its connected regions at a lower frequency oscillatory rhythm, resulting in a concomitant decay of the blood oxygenation level-dependent (BOLD) signal at the time scale of imaging^57^.

There were also several notable differences between states. State 2 exhibited a sparser set of strong functional connections to the rest of the subcortex. Further inspection revealed that the main differences involved basal ganglia substructures, as the bilateral putamen, anterior caudate and anterior globus pallidus all featured characteristically weak dFC with the left Vim in state 2. The basal ganglia are involved in the modulation of somatomotor cortical information processing^58^, and contribute to tremor generation^59^. Although ET has been primarily considered related to the cerebellum^2,12,13^, DBS of the posterior subthalamic area, an input source to the basal ganglia^58^, has shown effectiveness to treat ET^60^, and abnormal putamen activity^61,62^ and FC^63^ have been reported in ET. Thus, differential patterns of FC to the basal ganglia could relate to tremor symptomatology.

State 2 was also the one displaying the strongest anticorrelations between the left Vim and most of the cerebellum. This particularly involved the bilateral cerebellum lobules III and IV/V, as well as vermis III and vermis IV/V, which jointly contribute to the cerebellar sensorimotor component^64–66^. In addition, there was specifically more positive dFC to left cerebellum crus II and vermis VIII. The former is functionally related to the default mode network^64^, and a recent meta-analysis showed its specific roles in social mentalizing and emotional self-experiences^67^. As for Vermis VIII, its FC to the default mode, executive control and somatomotor networks was stronger in Parkinson’s disease patients suffering from visuospatial disorder on top of motor impairments^68^. Interestingly, we note that state 2 also stood out by its anticorrelations with parahippocampal and extrastriate visual areas, as well as with hippocampal subparts, which all contribute to visuospatial processing, and locomotor monitoring towards the local and distant environment^69^, relevant in ET patients.

Another intriguing feature of left Vim-to-cortex FC in the context of sensorimotor activity was the fact that states 1 and 3 showed only little correlated activity with primary sensorimotor areas, while the opposite was seen in state 2. In a recent study, Sharifi et al.^70^ studied cortico-muscular coherence (CMC) through coupled EEG and EMG recordings, in patients with ET and HCs asked to voluntarily mimic tremor. They showed that in both groups, CMC could be reliably detected and was modulated by performing a cognitive task in parallel; thus, non-tremor networks were posited to exert an interfering influence. Additionally, when tracking CMC changes with a sliding-window approach (30 s windows), there were dynamic fluctuations that could not be attributed solely to changes in tremor signal-to-noise ratio (SNR) or to task settings. The present work shows that motor cortical activity does reconfigure with time, given its evolving functional interplay with the left Vim, both in HCs and in patients with ET. Transitions between the expression of the more similar states 1 and 3, and state 2, may thus be hypothesized to capture fluctuating interfering influences from non-tremor networks, which would occur at rest and manifest in task settings too.

### Interactions with specific networks hinder or benefit clinical recovery

Interestingly, we found that temporal occurrences of states 1 and 3 prior to radiosurgical thalamotomy correlated with clinical recovery: indeed, more frequently expressing state 3 before the intervention associated with more pronounced upper limb tremor improvement, and when assessing the clinical predictive potential of the balance between these two states in a follow-up analysis, we observed that greater clinical recovery was in fact tied to a balance more geared towards state 3 expression.

**Figure 4:**
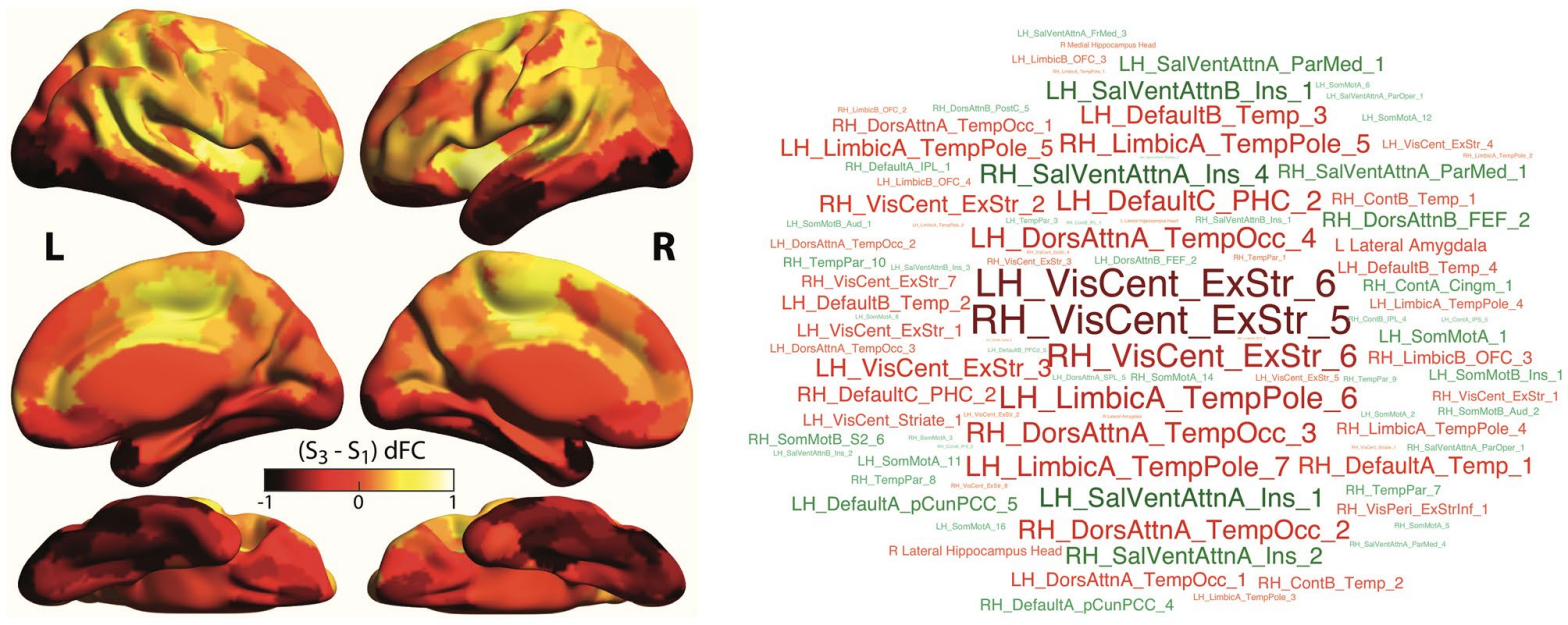
State 3 − state 1 dynamic functional connectivity differences. Differences in dFC of the left Vim with cortical areas are visualized as a surface plot (**A**), where dark/light tones highlight stronger dFC in state 1/3, and as a word cloud (**B**), where red/green highlights stronger dFC in state 1/3.

Given this antagonistic relationship, a natural question pertains to the differences in left Vim dFC between states 1 and 3. Accordingly, we inspected the state 3 − state 1 dFC differences (Fig. 4) and observed that more pronounced correlations between the left Vim and extrastriate central visual areas, temporal regions (from the temporal pole or occipito-temporal cortex) and the parahippocampal gyrus were particularly detrimental to clinical recovery (positive-valued in state 1 while weak in state 3). This set of regions constitute what has been referred by some as the *parieto-medial temporal pathway* for visuospatial processing^69^, which has been resolved in humans through RS FC analysis^71^. The relevance of the visuospatial circuitry in ET has become increasingly appreciated from the voxel-based morphometry^72^, SBM^30^, task-based fMRI^73^ and RS fMRI^35^ forefronts. Our results further contribute to this line of research and identify a more specific pathway whose intermittent functional coupling with the left Vim may be detrimental to tremor recovery following thalamotomy.

On the other hand, greater dFC to the insula, precuneus/posterior cingulate cortex and frontal eye fields was beneficial to clinical recovery (positive-valued in state 3 while weak in state 1). These regions are all part of higher-order functional networks. Thus, it could be that spontaneous “decoupling” of the tremor network at rest, through interactions of the left Vim with other interfering high-order networks, is beneficial to recovery following thalamotomy.

In addition to the above, we also observed that in ET patients before radiosurgical thalamotomy, a smaller standard deviation of spatial similarity to state 3 over time (that is, lower variability in state 3 expression) correlated with better clinical recovery. In other words, ET patients will improve more not only when they express state 3 more frequently before the intervention, but also when the expressed pattern of functional connectivity with the left Vim does not change over the course of time. Temporal occurrences of dFC states and spatial variability in their expression likely involve different underlying mechanisms: in the former case, a large-scale transition must occur from other configurations, and recent control theory studies have revealed how this can be achieved from the activation of only few structurally densely connected regions that can control the whole brain circuitry^74,75^. In the latter case, cross-regional interactions are more subtly modified across successive instances of state expression, which could for example be caused by external network perturbations.

In addition to a direct effect of variability in state 3 expression, we also resolved a significant interaction with ET familial history, so that less variable state 3 expression before intervention would not be as beneficial to patients with a family history of ET. Such a family history, as well as alcohol sensitivity, are more often present in early-onset ET patients, while tremor progresses faster in late-onset patients, leading to their dissociation into two distinct clinical sub-groups^7^. Our results position spatial variability in state expression as a functional brain correlate of these clinical differences. They also demonstrate the importance of an exhaustive assessment of sources of heterogeneity across ET patients, which may be clinical as in the present study, but also kinematic: indeed, Merchant and colleagues recently performed kinematic testing on a set of ET patients prior to Vim DBS and were able to predict which patients would show early tolerance to DBS stimulation^76.^

In fact, the clinical impact of the balance between states 3 and 1 expression was also seen as an interaction, with the MR signature volume one year after thalamotomy. This further demonstrates that to extract potentially predictive markers of clinical recovery, it is important to consider as many involved sources of variance as possible. In the case of thalamotomy performed by radiosurgery, the outcome itself may be related to factors such as individual sensitivity to radiation^21^ or specificity in the location of the relevant brain substructures^77^, thus yielding different individual radiobiological effects.

This latter point has been the subject of intense investigations: in a prospective DBS study, direct targeting of the dentato-rubro-thalamic tract yielded tremor improvement^78^, evidencing the benefits of targeting more mechanistically relevant structures. On a set of HCs, Middlebrooks et al.^79^ showed that the voxels targeted by stereotactic coordinates were mixed between being structurally connected to the primary motor cortex and supplementary motor area/premotor cortex, in a way that largely varied across subjects. Through diffusion MRI, several DBS studies also put forward candidate areas to which the volume of tissue activated (VTA) should be structurally connected to optimize interventional efficacy, including the connected area to the contralateral dentate nucleus^16,^ the supplementary motor area and premotor cortex^80^, or the primary motor cortex^81^. Purrer et al.^82^ showed that the MRgFUS lesion induced through Vim targeting only had 4% overlap with the Vim on average, but 43% with the cerebello-thalamic tract. In another study, the larger the fraction of lesioned voxels structurally connected to the precentral gyrus, the better the clinical recovery^83^.

Collectively, these reports highlight the limitations of relying on stereotactic coordinates and suggest that to better extract potential pre-intervention markers of tremor arrest, the targeting of a specific tract or thalamic subregion, via dedicated pre-thalamotomy imaging, may be a more suitable option. Ferreira and colleagues^84^ directly compared stereotactic Vim localization to the centroid of the volume of intersection between the thalamus and the cerebello-dentato-rubro-thalamo-cortical tract, reconstructed with probabilistic tractography. They showed marked differences in the associated coordinates, confirming the relevance of more advanced targeting approaches. While they also verified that tracking target volumes, head volume, and head movement exerted no significant impact on localization and demonstrated the reliability of extracted coordinates in a test–retest dataset, in another study aiming at segmenting thalamic voxels as a function of their structural connectivity, Bertino et al.^85^ revealed a significant impact of the specific methodology at play, including the type of algorithm used for tractography and the decision rule to assign a voxel. They also showed an impact of the parameters of the diffusion data (multi-shell versus single-shell, diffusion and angular resolutions), and evidenced marked inter-subject variability (> 2 mm) in the center of gravity of the thalamic cluster associated to some targets, whose origin could be due to technical reasons. In summary, while promising, technical hurdles remain to be overcome and further validation and harmonization of procedures must be conducted before stereotactic targeting can safely be replaced.

### Dynamic functional renormalization, but morphometric heterogenization upon thalamotomy

In terms of left Vim dFC, renormalization upon thalamotomy was observed in two ways (Fig. 2B). First, stability in the expression of all three states tended to increase upon intervention, significantly so for state 1. Second, the balance in expression of the three states following the intervention also evolved back towards that seen in HCs, and even overshot it, potentially reflecting a compensatory mechanism.

Contrarily to our expectations, however, we did not observe any renormalization when quantifying the evolution of whole-brain SBM properties upon intervention. In fact, the similarity of ET morphometric profiles to the HC distribution decreased even further upon thalamotomy in all regions (Fig. 3A). Further inspection revealed that mean values were unchanged upon thalamotomy. However, there were significant increases in CT and MC variance, and a global decrease of cross-property covariance, thus denoting morphometric heterogenization.

To explain this apparent discrepancy, we quantified the correlation between the log-likelihood of a morphometric profile to be issued from the HC distribution (*i.e.*, morphometric similarity to HCs) and mean spatial similarity in state expression (*i.e.*, dynamic functional similarity to HCs), before and after thalamotomy. To our surprise, we observed marked anti-correlations, at baseline, between states 2 and 3 (but not state 1) and the whole brain, which disappeared following thalamotomy (Fig. 3B, Supplementary Fig. 4).

**Figure 5:**
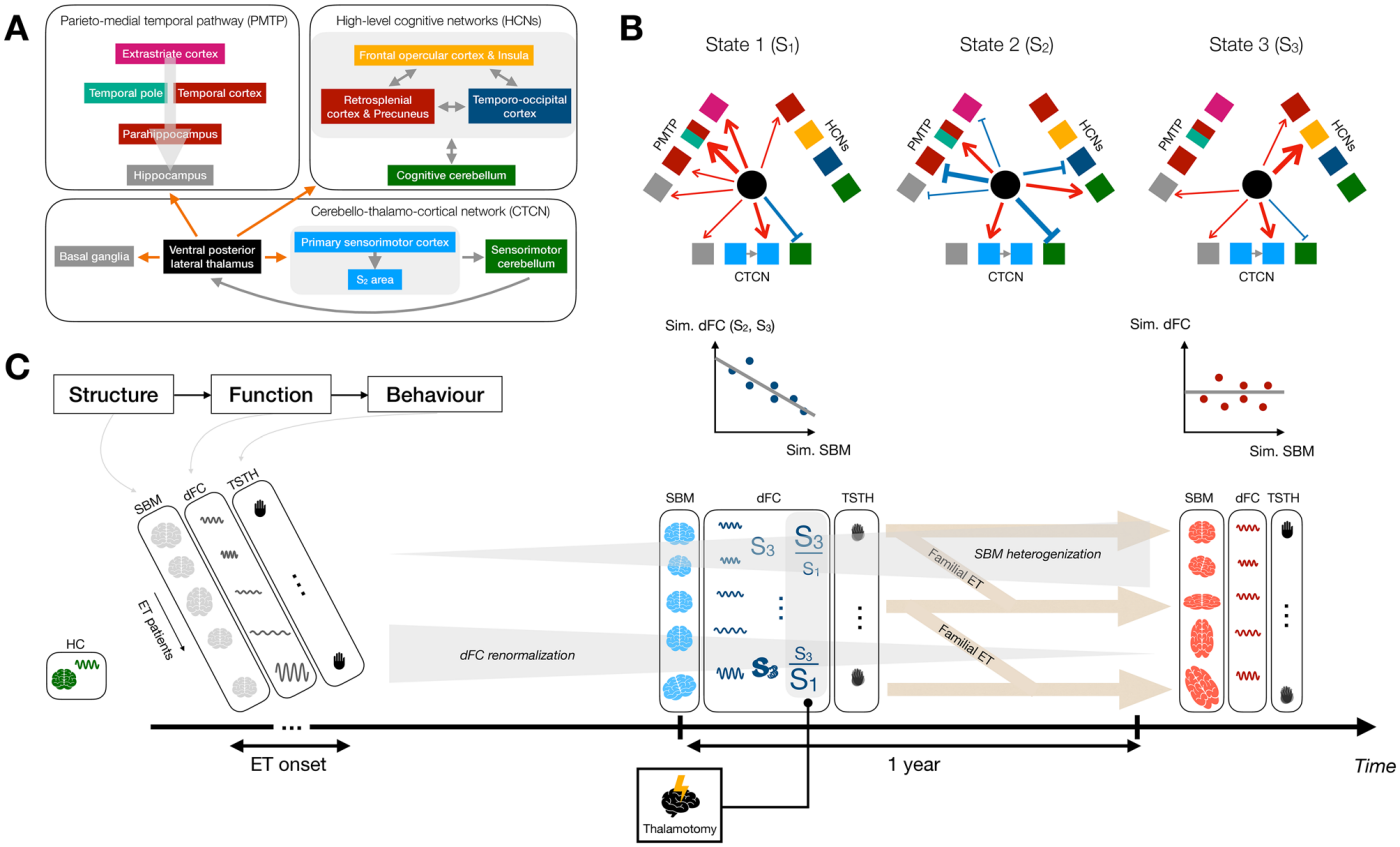
Summary of results. (**A**) The ventral posterior lateral thalamus (used as a seed in our dFC analyses) functionally interacts with the basal ganglia and sensorimotor regions within the cerebello-thalamo-cortical network (CTCN), with areas from the parieto-medial temporal pathway (PMTP), and with high-level cognitive networks (HCNs). (**B**) These functional interactions vary over time across three dFC states: in particular, state 1 (S_1_) showcases strong positive-valued dFC to the whole PMTP, state 2 (S_2_) is characterized by strong anticorrelations with the parahippocampus within the PMTP and the sensorimotor cerebellum, and state 3 (S_3_) displays strong positive-valued dFC to the salience network. The width of the arrows is qualitatively proportional to the intensity of dFC. (**C**) Brain structure, here quantified by surface-based morphometry (SBM), is the substrate over which brain function (studied through dFC analysis) occurs. The latter itself drives behavior (in the present case, tremor quantified via the Tremor Score on Treated Hand [TSTH]). When essential tremor (ET) declares itself (with variable onset age), each patient has characteristic morphometric and functional activity profiles, without any tremor. As the disease unfolds, SBM features become gradually more distinct from those of healthy controls (HCs, denoted in green on the left), while dFC renormalizes. At the time of thalamotomy, there is an anticorrelation between dFC and SBM similarity to HCs. The patients who, before intervention, express S_3_ with greater spatial stability and exhibit an S_3_/S_1_ balance more geared towards the former state, are the ones whose tremor is reduced the most 1 year after thalamotomy. Increased stability in S_3_ expression will associate with greater clinical improvement less strongly if a familial ET history is present, while the S_3_/S_1_ occurrence ratio impact is modulated by the MR signature. 1 year after thalamotomy, the anticorrelation between dFC and SBM similarity to HCs has vanished.

Considering these intriguing results, our personal view is that brain function in dedicated areas induces tremor. As such, “healthy” dFC states can be seen as the target towards which an individual patient should tend to minimize tremor symptomatology. Brain structure, partly characterized by morphometric properties, is the underlying scaffold over which functional signals propagate. Essential tremor patients who resemble HCs in their whole-brain morphometric attributes, but suffer from local impairments within the tremor network, may not be able to express HC-like dFC states owing to these local alterations. To achieve such expression, further reconfigurations of the whole-brain structural circuitry are required. Thus, the patients who undergo such reconfigurations the most (by this mean becoming least like HCs morphometry-wise) are also the ones who can express the dFC states most similar to HCs, resulting in the observed anticorrelations. These anticorrelations are only present for states 2 and 3, which appeared non-detrimental (state 2) or beneficial (state 3) to clinical recovery in our analyses, as these are the desired endpoints to limit tremor. After thalamotomy, these relationships disappear because further extensive changes are induced in all subjects, in a way that is also heterogeneous. Figure 5 summarizes our results in the context of this hypothesis, which should be validated in future work.

### Technical outlook, limitations, and future perspectives

In this work, we analyzed the data from ET patients in terms of similarity to a pool of matched HCs. This strategy is particularly suitable to the study of ET for two reasons: first, it enables to investigate renormalization upon thalamotomy more directly as opposed to a three-group comparison (*i.e.,* HC versus ET_pre_ versus ET_post_). Second, it yields distinct, potentially more relevant features in the context of a clinical disorder. We were inspired by recent work that successfully leveraged a conceptually related approach: in a DBS study of ET, Al-Fatly and colleagues^86^ quantified structural and functional connectivity to the VTA, on a population of ET patients, using normative maps. To create a so-called “R-map”, for each voxel, they correlated the obtained values (i.e., connectivity to the VTA) with clinical improvement, so that the resulting pattern has large positive weights for the voxels that, when connected to the VTA, promote clinical recovery. Out-of-sample data could then be compared to this R-map to estimate clinical improvement, and prediction was significant when using both structural and functional connectivity information. In the present study, we showed that a similarity-based approach also has potential in the context of morphometric and dynamic functional connectivity data.

In future work, it will be necessary to validate our findings on a larger cohort of subjects. Our sample size (18 per ET group for dFC analysis, 34 per ET group for SBM analysis) was within the range of contemporary clinical neuroimaging studies (median of 12.5 for functional studies and 24 for structural studies for articles published between 2017 and 2018)^87^, and was primarily dictated by the difficulty in enrolling patients satisfying the criteria to be part of our cohort (*i.e.*, suffering from drug-resistant ET with clinical and neuroimaging [structural and functional MRI] assessments before and 1 year after left Vim stereotactic radiosurgical thalamotomy). For dFC analysis, our stringent criteria to retain scans to ensure a whole-brain field of view and prevent spurious head movement-related effects (see Materials and methods) also largely contributed. Low sample size can exert often under-appreciated deleterious effects including a greater risk that a significant finding does not reflect a true effect^88^, and the inflation of effect sizes found in significant outcomes^89^. To mitigate the first issue as much as possible, on top of applying Bonferroni correction to our analyses, we set to use a significance level of 0.01 rather than the usual 0.05. The second can be seen in our results through the large 99% confidence intervals associated to the reported effect sizes; however, note that for our main results (balance in state 3/state 1 temporal occurrences and standard deviation of spatial similarity in state 3 expression correlating with clinical recovery), zero is not part of the confidence interval.

Directly related to our small sample size is the potential confounding impact of heterogeneity across ET patients. To counter this, we implemented several analytical strategies: first, when assessing whether thalamotomy exerted significant effects on metrics reflective of temporal occurrences and spatial similarity to the extracted dFC states, subject heterogeneity was explicitly modeled by means of a random effect (*i.e.*, one intercept term per subject), and was thus factored out, not impacting the provided fixed group effect results (that is, the pre- *versus* post-thalamotomy contrast). Second, when probing the associations between pre-interventional metric values and clinical recovery, we included interactions between the metric at hand and all the clinical variables to our disposal reflective of heterogeneity across patients, as well as the MR signature volume, potentially reflective of heterogeneity in genetic background or in exact localization of the Vim. Third, when considering morphometric data, we assessed the likelihood of three-dimensional (cortical thickness, surface area, mean curvature) data points from ET patients to be issued from the distribution of HC data points, modeled as a multivariate Gaussian. The resulting log-likelihood value was low if the data point at hand was dissimilar from the HCs distribution, regardless of how exactly it differed (*e.g.*, CT may have been strongly altered in one patient, while SA was in another, but both cases could have yielded the same log-likelihood value); thus, heterogeneity across patients was directly integrated in our mathematical framework. Previous work of ours has demonstrated the benefits of this approach in revealing otherwise overshadowed links between SBM features and the extent of upper limb tremor in ET patients^36^.

Our seed-based dFC analysis was conducted using the Tian scale 3 subcortical atlas^45^. Despite an exhaustive state-of-the-art segmentation of the subcortex, only the ventral posterior lateral thalamus was available as a seed, not the Vim per se, which covers its ventral portion only^90^. To confirm that this did not impact our findings, we extracted dFC states from our population of HCs when, in lieu of the Tian atlas region, using the volume of intersection between the thalamus and the cerebello-dentato-rubro-thalamic tract inferred from the atlas provided by Basile and colleagues^91^ as a seed. The resulting states strongly resembled the original ones (spatial correlation values of 0.85, 0.81 and 0.83 for states 1 to 3; see Supplementary Fig. 5).

Another potentially worrying factor is the fact that the region taken as our seed in dFC analysis was effectively lesioned upon thalamotomy, leading to the MR signature discussed above. One may thus wonder if keeping it for investigations after 1 year makes sense. There are different subregions within the MR signature, and only the area lying within the 4-mm diameter isocenter of irradiation is believed to undergo necrosis^92^. The Tian atlas parcel in which this targeted area lies, the left ventral posterior lateral thalamus, is made of 784 voxels at a resolution of 1 mm^3^. Approximating it as a sphere for simplicity, this amounts to a radius of 13.68 voxels, or 13.68 mm. In other words, the volume of necrosed tissue only makes roughly 2.14% of the region volume. Thus, it is reasonable to expect that an averaging of these voxels still captures a meaningful signal after thalamotomy. Of course, changes are also induced outside of the necrotic area itself. While the exact mechanisms at play remain to be elucidated, it has been suggested that glial cells may be particularly sensitive to irradiation and undergo a large turnover upon thalamotomy, leading to pronounced changes in the environment of the neurons^92^.

Unlike dFC analysis, our SBM investigations made use of a more compact atlas of only 87 brain regions. Thus, one may think that cross-modality assessments are complicated, as separate correlational assessments in each brain region then cannot be conducted. However, this is in fact not the case, as we focus on the study of similarity to HCs: as such, we derived dynamic functional connectivity states from the population of healthy controls, and then quantified average similarity to these states for frames where they were expressed in ET patients, gathering similarity data of size *K* times *S* (number of ET samples in a group). For morphometric analyses, in each region, we quantified the log-likelihood of SBM profiles to be issued from the distribution of HCs. We thus gathered data of size *P* times *S*. Both sets of results can then be correlated across subjects and examined for each of the states as a histogram showing regional distribution of similarity values (see Fig. 3B). The implementation of other cross-modality analytical strategies, potentially relying on the same atlas in the SBM and dFC forefronts, shall be an interesting avenue for future work. However, it will come with the need to develop regional measures reflective of dFC, while connection-wise or state features are classically extracted^33^.

Other notable ideas for future work include the assessment of right Vim dFC, to assess whether thalamotomy also induces modulations to its interactions with the rest of the brain, and the application of control theory tools to the brain circuitry involved in ET, so that potential drivers of state transitions can be put forward. Finally, if one wishes to further understand the links between brain structure and function, and how they may be altered upon thalamotomy, morphometric data (indicative of local structure within specific regions) should be complemented by diffusion MRI. By this mean, the physical wiring between brain regions could refine cross-modality assessments.

### Supplementary Information

Supplementary Information.

Supplementary Figure 1.

Supplementary Figure 2.

Supplementary Figure 3.

Supplementary Figure 4.

Supplementary Figure 5.

Supplementary Table 1.

Supplementary Table 2.

## Data Availability

The data that support the findings of this study are available from the corresponding author upon reasonable request.
