# Supplementary material for: Dynamic functional changes upon thalamotomy in essential tremor depend on baseline brain morphometry
Source: Sci Rep. 2024 Jan 31;14:2605. doi: 10.1038/s41598-024-52410-y (PMC10831051; doi:10.1038/s41598-024-52410-y)
Supplement: Supplementary file 1 — Supplementary Information. [file 41598_2024_52410_MOESM1_ESM.docx]

**Supplementary information**

**Materials and Methods**

**Subjects**

Only patients meeting the following inclusion criteria were analysed: confirmed ET diagnosis, able to give formal approval, [drug](https://www.sciencedirect.com/topics/medicine-and-dentistry/chemotherapeutic-agent) resistance (propranolol or primidone) after adequate trials, age between 18 and 80 years, and targeted thalamic area apparent on pretherapeutic magnetic resonance imaging. Patients with mixed or Parkinsonian tremor were excluded, as were those with previous contralateral radiosurgical thalamotomy, epilepsy, brain tumours, or stroke. The main indications for stereotactic radiosurgical thalamotomy were medical comorbidities, advanced age, or patient refusal of deep brain stimulation.

**Data acquisition**

Native T1-weighted structural images were acquired for all subjects on the same head-only 3T machine (SIEMENS SKYRA, Munich, Germany, 32-channel receive-only phase-array head coil). For ET patients, data was collected both at baseline and one year after thalamotomy. The acquisition parameters were as follows: TR/TE=2300/2.98 ms, isotropic voxels of 1 mm^3^, 160 slices. Scanning was performed in a drug-naïve state (drugs having been stopped at least 3 days beforehand). One year after thalamotomy, the scan included a sequence with Gadolinium injection, to help better distinguish and delineate the corresponding MR signature and its volume (see below).

RS fMRI scans were acquired for the same subjects (both before and after thalamotomy for ET patients), using the same machine and the following parameters: TR/TE=3300/30 ms, flip angle=90°, voxel size 4 mm^3^, 300 volumes acquired per ET patient (210 for HCs), 46 interleaved axial slices. Participants were asked to relax with their eyes closed, without falling asleep or engaging in cognitive or motor tasks.

**Extraction of morphometric features**

Following linear registration to Montreal Neurological Institute space and bias field removal, the image at hand was skull-stripped,^1^ and voxels were classified as belonging to white matter or to another tissue category based on intensity and direct neighborhood. Following the separation of hemispheres and the removal of the cerebellum and subcortex, the interface between the white and gray matters and the pial surface were located. Local estimates of CT, SA and MC were then extracted.^2^ These voxel-wise measurements were eventually converted into P_cort_ regional values per morphometric measure, using the Desikan-Killiany atlas.^3^ For each cortical brain region, a three-dimensional data point was thus obtained per subject. C.T. checked the quality and consistency of intermediate and final output files for each scan.

**Preprocessing of morphometric features**

For each region, each morphometric feature (CT, SA or MC for cortical areas and volume for non-cortical ones) was modelled as:

$M_{i,s}=\beta_{0}+\beta_{1}A_{s}+\beta_{2}G_{s}+\beta_{3}V_{s}+\sum_{k=1}^{2} \beta_{3+k}I\left[ C_{k+1} \right]_{s}A_{s}+\beta_{5+k}I\left[ C_{k+1} \right]_{s}G_{s}+\beta_{7+k}I\left[ C_{k+1} \right]_{s}V_{s}+b_{0,s}+\epsilon_{i,s}$.

In the above, *M_i_*_,_*_s_* is the morphometric feature’s value for region *i* and subject *s*. The model includes an intercept (*β*_0_ coefficient) and considers the individual impacts of age (*A_s_*, *β*_1_), gender (*G_s_*, male=0, female=1, *β*_2_) and total grey matter volume (*V_s_*, *β*_3_).

Regarding the impact of group (*C*_1_ to C_3_ for HC, ET_pre_ and ET_post_, respectively), *I*[*C_k_*]*_s_* is a dummy variable encoding whether subject *s* belongs to group *C_k_*. Because of the presence of an intercept term in the model, only two dummy variables are needed to account for three groups. We model the interactions between group and the three confounds (*i.e.*, we enable distinct extents of confounding impacts across groups, as summarized by coefficients *β*_4_ to *β*_9_).

The term *b*_0,_*_s_* is the random effect for subject *s* (a subject-specific intercept), which follows a normal distribution with mean 0 and standard deviation $\sigma_{b}$. Finally, $\epsilon_{i,s}$ is the error term for region *i* and subject *s*, following a normal distribution with mean 0 and standard deviation $\sigma$.

The residuals of the model (including random effects, as well as group effects since they were not explicitly modelled) were used for all subsequent analyses.

**Computation of morphometric similarity to HCs**

For cortical brain regions, we considered three-dimensional (CT, SA, MC) data points for each subject. For non-cortical brain regions, we considered one-dimensional data points (regional volume), where univariate Gaussians are fitted and only two parameters characterize a distribution (the mean and standard deviation).

For each region, the HC data was modelled by a multivariate normal distribution with mean vector $\boldsymbol{\mu}$ and covariance matrix $\boldsymbol{\Sigma}$, which jointly describes the variance for each morphometric property (diagonal elements), and the covariance across them (off-diagonal elements; see ^26^ for further mathematical details). The best mean vector and covariance matrix estimators $\hat{\boldsymbol{\mu}}$ and $\hat{\boldsymbol{\Sigma}}$, in the log-likelihood sense, are trivially the data’s arithmetic mean and covariance matrix.

The log-likelihood (LL) of a data point ***x*** (which represents the morphometric estimate[s] of a given subject before or after thalamotomy) to be issued from this distribution can then be computed as:

$\mathcal{L}\mathcal{L}_{\mathcal{N}}\left( \boldsymbol{x}|\hat{\boldsymbol{\mu}},\hat{\boldsymbol{\Sigma}} \right)=-\frac{1}{2}\ln\left( \sqrt{2\pi\left| \hat{\boldsymbol{\Sigma}} \right|} \right)+\left( \boldsymbol{x}-\hat{\boldsymbol{\mu}} \right)^{T}{\hat{\boldsymbol{\Sigma}}}^{-1}\left( \boldsymbol{x}-\hat{\boldsymbol{\mu}} \right)$.

**Results**

**Supplementary SBM analyses**

**Individual mean and (co)variance coefficients**

To further characterize ET_pre_ *versus* ET_post_ differences, we compared both groups at the level of individual mean and (co)variance coefficients. There were no differences in mean values across groups, but CT variance increased upon thalamotomy in the bilateral fusiform gyrus (*p*=0.002 and *p*=0.003, respectively left and right sides), left lateral orbitofrontal cortex (*p*=0.002), precentral gyrus (*p*=0.003), and right lingual cortex (*p*=0). MC variance also increased in the bilateral fusiform gyrus (*p*=0.008 and *p*=0) and left insula (*p*=0). Covariance between CT and MC decreased in the left fusiform gyrus (*p*=0.003), lateral orbitofrontal cortex (*p*=0.012) and right superior temporal cortex (*p*=0.01). Covariance between CT and SA decreased as well in the left precentral gyrus (*p*=0.006). However, covariance between SA and MC increased in the bilateral fusiform gyrus (*p*=0.009 and *p*=0.013). Variance in subcortical volume increased in the bilateral hippocampus (*p*=0 and *p*=0.009). **Supplementary Figure 3** visually illustrates all these changes.

**Analysis on only ET patients considered in dFC investigations**

When examining how morphometric similarity to HCs would be affected by only considering the same set of patients as in dFC analyses (that is, 23 out of the 34 available ones), the average log-likelihood across subjects remained consistently larger before than after thalamotomy, significantly so in the left fusiform gyrus (*z*=-3.53, *p*=0.037, η^2^=0.54), bilateral parahippocampal gyrus (*z*=-4.19, *p*=0.002, η^2^=0.76 and *z*=-4.07, *p*=0.004, η^2^=0.72, respectively left and right sides), and left hippocampus (*z*=-3.72, *p*=0.018, η^2^=0.61). All these regions were part of the ones unraveled by the analyses conducted on the full set of ET patients. Thus, we conclude that the smaller statistical power inherent to this supplementary analysis explains the more restricted set of significant regions, and that our main results on the full population of ET patients accurately represent what can be expected at the level of the patients for whom dFC analysis was conducted.

In terms of individual mean and (co)variance coefficients, similarly to the main analyses, CT variance increased in the left fusiform gyrus (*p*=0.011), and so did variance in left hippocampal volume (*p*=0.002). Only one new significant difference was detected: an increase in SA variance in the left parahippocampal gyrus (*p*=0.009).

**Supplementary investigations on links with clinical recovery**

As a supplementary sanity check, we probed the unraveled associations between pre-thalamotomy dFC metrics and clinical recovery using only metric, MR signature volume, age and gender as regressors, thus not including clinical variables reflective of heterogeneity (presence of a familial history of ET, duration of symptoms, head tremor).

For the standard deviation of spatial similarity to state 3, our main analyses revealed a significant metric effect (*t*_6_=-4.24, *p*=0.005, Cohen’s *d*=-1.00±0.91), a significant interaction with family history of ET (*t*_6_=4.14, *p*=0.006, Cohen’s *d*=0.98±0.91), and a significant effect of head tremor (*t*_6_=-4.15, *p*=0.006, Cohen’s *d*=-0.98±0.91). With our restricted set of regressors, the metric effect became non-significant, although it kept the same direction (*t*_12_=-2.00, *p*=0.069, Cohen’s *d*=-0.47±0.87). This is because the restricted set of regressors does not allow to disentangle the metric effect, and its interaction with familial ET history. In other words, it shows the importance of explicitly accounting for this clinical factor of heterogeneity.

For temporal occurrences, the interaction found between state 3 and MR signature volume in our original analyses (*t*_6_=3.76, *p*=0.009, Cohen’s *d*=0.89±0.9) remained significant (*t*_12_= 3.12, *p*= 0.009, Cohen’s *d*=0.73±0.89). For state 1, where the interaction was not significant but showed an opposite effect direction (*t*_6_=-2.32, *p*=0.059, Cohen’s *d*=-0.55±0.87), significance increased, but still did not pass our significance threshold of 0.01 (*t*_12_=-2.73, *p*= 0.018, Cohen’s *d*=-0.64±0.88). The significant interaction between MR signature volume and the State 3 – State 1 balance (*t*_6_=4.6, *p*=0.004, Cohen’s *d*=1.08±0.92) was also present (*t*_12_=3.16, *p*=0.008, Cohen’s *d*= 0.75±0.89). In summary, our results regarding the temporal occurrences of states 1 and 3 are robust to adjustments in the employed general linear model and remain significant regardless of whether clinical sources of cross-patient heterogeneity are modeled or not.

**References**

# Ségonne F, Dale AM, Busa E, Glessner M, Salat D, *et al*. A hybrid approach to the skull stripping problem in MRI. *Neuroimage*. 2004;22(3):1060-1075.

# Fischl B, Dale AM. Measuring the thickness of the human cerebral cortex from magnetic resonance images. *Proceedings of the National Academy of Sciences U.S.A.*. 2000;97(20):11050-11055.

# Desikan RS, Ségonne F, Fischl B, Quinn BT, Dickerson BC, *et al*. An automated labeling system for subdividing the human cerebral cortex on MRI scans into gyral based regions of interest. *Neuroimage*. 2006;31(3):968-980.
