# Supplementary figures and images for: Dynamic functional changes upon thalamotomy in essential tremor depend on baseline brain morphometry
Source: Sci Rep. 2024 Jan 31;14:2605. doi: 10.1038/s41598-024-52410-y (PMC10831051; doi:10.1038/s41598-024-52410-y)

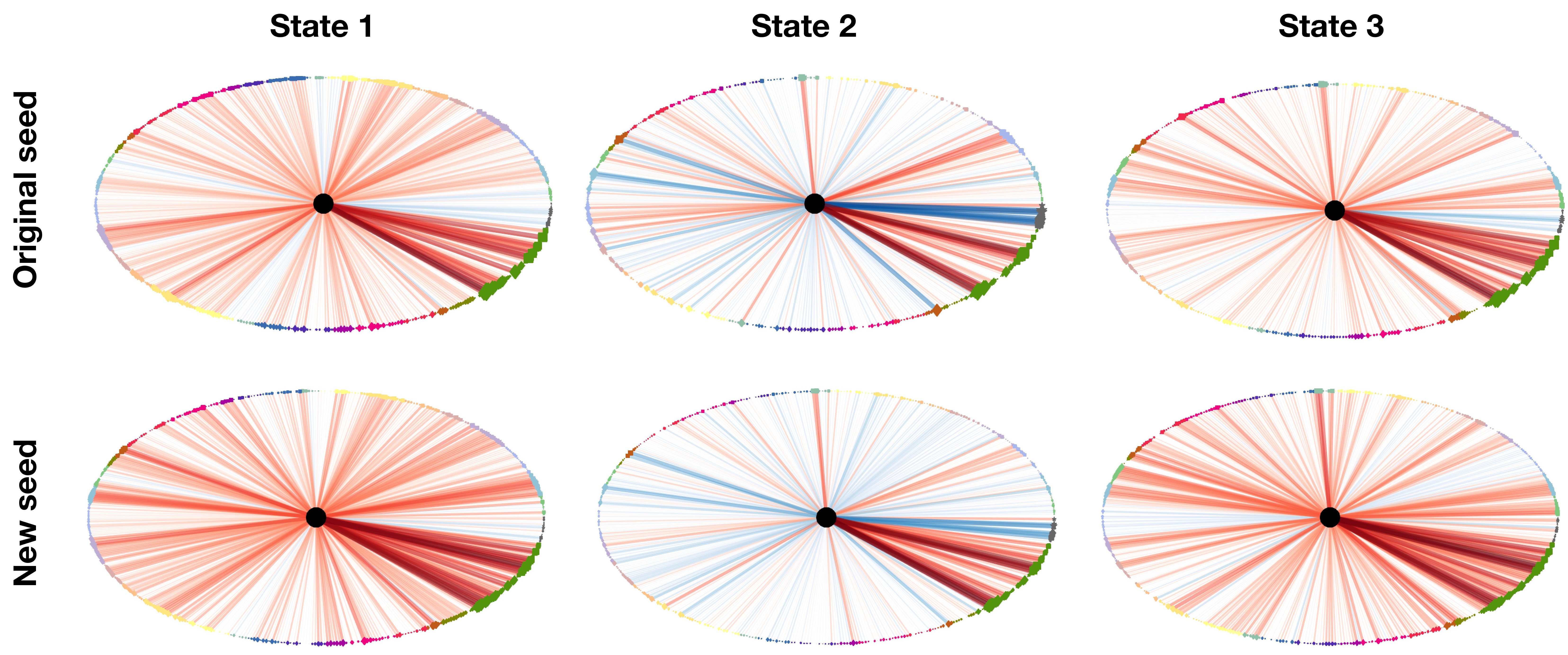

Supplement: Supplementary file 6 — Supplementary Figure 5. [file 41598_2024_52410_MOESM6_ESM.jpg]
